# A role for the *tfs3* ICE-encoded type IV secretion system in pro-inflammatory signalling by the *Helicobacter pylori* Ser/Thr kinase, CtkA

**DOI:** 10.1371/journal.pone.0182144

**Published:** 2017-07-28

**Authors:** Maher N. Alandiyjany, Nicola J. Croxall, Jane I. Grove, Robin M. Delahay

**Affiliations:** NIHR Nottingham Digestive Diseases Biomedical Research Unit, Nottingham University Hospitals NHS Trust and University of Nottingham, Nottingham, United Kingdom; National Cancer Center, JAPAN

## Abstract

Two distinct type IV secretion systems (T4SSs) can be identified in certain *Helicobacter pylori* strains, encoded on mobile genetic elements termed *tfs3* and *tfs4*. Although their function remains unknown, both have been implicated in clinical outcomes of *H*. *pylori* infection. Here we provide evidence that the Tfs3 T4SS is required for activity of the pro-inflammatory Ser/Thr kinase protein, CtkA, in a gastric epithelial cell infection model. Previously, purified recombinant CtkA protein has been shown to upregulate NF-kappaB signalling and induce TNF-alpha and IL-8 cytokine secretion from cultured macrophages suggesting that it may potentiate the *H*. *pylori*-mediated inflammatory response. In this study, we show that CtkA expressed from its native host, *H*. *pylori* has a similar capacity for stimulation of a pro-inflammatory response from gastric epithelial cells. CtkA interaction was found to be dependent upon a complement of *tfs3* T4SS genes, but independent of the T4SSs encoded by either *tfs4* or the *cag* pathogenicity island. Moreover, the availability of CtkA for host cell interaction was shown to be conditional upon the carboxyl-terminus of CtkA, encoding a putative conserved secretion signal common to other variably encoded Tfs3 proteins. Collectively, our observations indicate a role for the Tfs3 T4SS in CtkA-mediated pro-inflammatory signalling by *H*. *pylori* and identify CtkA as a likely Tfs3 T4SS secretion substrate.

## Introduction

Persistent infection of the gastric mucosa of up to half of all humans with *Helicobacter pylori* is a predisposing factor for a range of severe gastroduodenal diseases including peptic ulcer disease and gastric cancer [1, 2]. Determination of a particular disease outcome is considered to involve a complex interplay between bacterial, host and environmental factors [3, 4]. Of the bacterial factors, a subset of surface-associated and secreted proteins are of prominent importance for defining the pathogenic potential of a particular infecting *H*. *pylori* strain and include the *cag* type IV secretion system (T4SS) and its secreted effector protein CagA, the vacuolating cytotoxin VacA and the adhesins BabA and SabA [4–10]. The variable activity of these virulence proteins has been associated with the extent of damage to the gastric mucosa and severity of the host inflammatory response. More recently, other *H*. *pylori* proteins such as Tipα [11] and cell translocating kinase A (CtkA) [12, 13] have also been described to induce pro-inflammatory responses from host cells. Whereas the *tipα* gene appears conserved in *H*. *pylori* genomes, the *ctkA* gene is considerably more variable in different geographic populations and comprises part of the subset of strain-specific genes that contributes to the extreme genetic diversity of *H*. *pylori* strains [12–19].

Perhaps the best studied of these latter proteins, CtkA, has been shown to be highly expressed in response to *H*. *pylori* interaction with the gerbil gastric mucosa in an *in vivo* study [20]. Work using recombinant CtkA protein (rCtkA) has also demonstrated dose-dependent induction of pro-inflammatory cytokine TNF-α and IL-8 secretion from cultured macrophage and mononuclear cells [12, 13] and pro-inflammatory and pro-apoptotic responses from mouse macrophages [21]. These studies suggest that CtkA may contribute to chronic gastric inflammation and *H*. *pylori* persistence, thereby increasing the risk of inflammation-associated outcomes such as atrophy and gastric cancer. Indeed, the gene encoding *ctkA* (*jhp0940* in reference *H*. *pylori* strain J99) has been reported to have a positive association with increased risk of gastric cancer in some populations [14] and decreased risk for duodenal ulcer [19].

Structural solution of C-terminally truncated rCtkA and further mechanistic studies identified it to be both a Ser/Thr and tyrosine kinase capable of auto-phosphorylation [12, 21]. Interestingly, rCtkA has a capacity to self-translocate across the membrane of cultured epithelial cells and is presumed to elicit host responses through direct engagement of intracellular signalling molecules.

A large number of strain-specific genes are now known to be encoded within complete or remnant transmissible genetic elements variously referred to as conjugative transposons (TnPZs) or self-transmissible genomic islands [22–24]. Two have been described, termed *tfs3* and *tfs4*, both of which comprise a full complement of genes encoding Vir-homologous T4SS assembly proteins in addition to a variable number of cargo genes of unknown function. The gene encoding the VirB4 ATPase component of the putative Tfs4 T4SS, *dupA*, has been associated with increased risk for duodenal ulcer in several geographically distinct patient populations [25] suggesting that activity of a Tfs4 T4SS may be important for clinical outcome. However, the functional activity of the Tfs4 T4SS remains undefined, although *dupA*+ strains have been reported to demonstrate both increased survival at low pH and upregulation of IL-8 secretion from gastric epithelial cells [26]. Upregulation of pro-inflammatory signalling has also been reported as a feature of certain strains bearing *tfs3* [23]. Further mechanistic studies have implicated the putative Tfs4 T4SS in transfer of the *tfs4* cluster [24], and identified catalytically active *tfs4*-encoded protein components such as the XerD integrase [24] and VirD2 relaxase [27] relevant to this function. These proteins are similarly encoded by *tfs3*, indicating that both elements may have a propensity for dissemination within the *H*. *pylori* population by a conjugative-like mechanism analogous to that of integrative and conjugative elements (ICEs) [27, 28]. Thus, gain or loss of *tfs3* and *tfs4* ICE-like clusters, each comprising 30–40 genes, could be anticipated to significantly contribute to the genetic diversity of *H*. *pylori* strains, providing a means for rapid augmentation or disposal of genotypic and phenotypic characteristics that may influence strain fitness and the nature of the interaction with its host.

The current known functional activity of CtkA, both with respect to its catalytic activity and interaction with cultured human cells, derives from the study of recombinant protein (rCtkA) overexpressed and purified from a heterologous bacterial host [12, 13]. However, whilst providing important mechanistic insight, such studies do not allow for assessment of CtkA activity in the context of *H*. *pylori* infection and have not been confirmed to reflect the activity of native CtkA protein. Therefore, in this study we aimed to develop an infection model to facilitate study of CtkA expressed from its native *H*. *pylori* host, and further, to determine factors important for its subsequent interaction with host eukaryotic cells. We show that *ctkA* is a variable but constituent component of the *tfs3* ICE and demonstrate that pro-inflammatory signalling by gastric epithelial cells in response to CtkA expressed from *H*. *pylori* requires both CtkA C-terminal sequence and the complement of Tfs3 T4SS genes.

## Materials and methods

Chemicals and reagents were obtained from Sigma-Aldrich unless otherwise stated.

### Bacterial strains, cell lines and growth conditions

Bacterial strains used in this study are described in S1 Table. *H*. *pylori* strains were cultured with minimal passage on Blood Agar Base No. 2 containing 5% (vol/vol) horse blood (Oxoid) or in 25 cm^2^ flasks in RPMI 1640 medium supplemented with 5% heat-inactivated fetal calf serum with shaking at 37°C in a microaerobic environment. *Escherichia coli* strains were grown at 37°C in Luria broth or agar. All media was supplemented with ampicillin (50–100 μg ml^-1^), kanamycin (50 μg ml^-1^) or chloramphenicol (30 μg ml^-1^) as required.

The human gastric epithelial MKN28 [29] and AGS cell lines (ATCC CRL-1739) were maintained in RPMI 1640 and F12-HAM medium respectively, supplemented with 10% heat-inactivated fetal calf serum and 2 mM L-glutamine (Invitrogen). Cell monolayers were grown at 37°C in a 5% CO_2_ humidified atmosphere. *H*. *pylori* strain 64 was isolated from human gastric biopsy specimens as described previously [30].

### Oligonucleotides

The oligonucleotides used in this study are listed in S2 Table.

### Sequence analyses

Genome sequences were retrieved from the NCBI database (http://www.ncbi.nlm.nih.gov/pubmed) from where BLASTP/N searches were also performed using default parameters. Multiple sequence alignments were performed using Clustal Omega (http://www.ebi.ac.uk/Tools/msa/clustalo/) and pairwise alignments using the EMBOSS Needle alignment tool (http://www.ebi.ac.uk/Tools/psa/emboss_needle/). Alignments were shaded using Boxshade version 3.3.1 (http://mobyle.pasteur.fr/cgi-bin/portal.py?#forms::boxshade).

### Molecular techniques

DNA manipulations were performed by standard techniques in *E*. *coli* strain XL1-Blue. Genomic DNA from *H*. *pylori* strains for use as PCR template was prepared after 48 h plate culture using a genomic DNA preparation kit (Sigma). Taq DNA polymerase (New England BioLabs) was used for PCR-based typing of *H*. *pylori* strains according to manufacturer’s recommendations using the oligonucleotide typing primer (‘TP-‘) pairs listed in S2 Table.

The *gsk-ctkA* or 3’ truncated *gsk-ctkA*_1-906_ inserts were amplified from strain J99 using Phusion DNA polymerase (New England BioLabs) and primers GSK-ctkANdF and ctkASR, then initially introduced into *Nde*I/*Sal*I sites of pSB14 by directional cloning to create plasmid pSB14-*gsk-ctkA*.

For construction of pGEM-*cagE*::*kan*, a partial *cagE* gene (*cagE*_257-1966_) was amplified from strain 26695 using the Expand High Fidelity PCR kit (Roche) with typing primers TP-cagEF/TP-cagER, then TA-cloned to pGEM-TEasy. Primers cagEiPXmR/cagEiPXhF were next used to remove a central segment of *cagE* by inverse PCR, at the same time introducing *Xma*I and *Xho*I sites into which a kanamycin resistance cassette, amplified with primers kanXmBF/kanXhR, was subsequently inserted. To generate pGEM-*cagE*::*gsk-ctkA-kan*, the *gsk-ctkA* insert fused to the *H*. *pylori flaA* promotor sequence in pSB14-*gsk-ctkA* was amplified using primers SBflaAXmF/apha3R, then cloned to *Xma*I/*Bam*HI sites of pGEM-*cagE*::*kan*. For pGEM-*cagE*::*gsk-ctkA*_1-906_-*kan*, insert was instead amplified using primers SBflaAXmF/ctkABR2, then cloned in the same way. For pGEM-*virB9*::*cat*, the *tfs3 virB9* gene was amplified from *H*. *pylori* strain AB5 using primers tfs3virB9F/tfs3virB9R, then TA-cloned to pGEM-TEasy. The inverse PCR primer pair tfs3B9iPXmR/tfs3B9iPXhF was then used to introduce central *Xma*I and *Xho*I sites into which a chloramphenicol resistance cassette was cloned. pGEM-based constructs were introduced into *H*. *pylori* strains using either electroporation or natural transformation.

### *In vitro* culture and secretion experiments

MKN28 or AGS human gastric epithelial cells were seeded into 24 wells at 5 x 10^4^ cells/well 24 h before experiments for assessment of cytokine, or 25 cm^2^ flasks at 7 x 10^5^ cells/flask for secretion experiments. Relevant *H*. *pylori* strains were added to cell monolayers at a multiplicity of infection (MOI) of 50:1 unless otherwise stated. Sampled cell culture supernatants were assessed for IL-8 or TNF-α levels using Human IL-8 CytoSet or Human TNF-α ELISA kits (Invitrogen). Cell culture supernatants for protein analysis were 0.2 μM filtered prior to 5 min incubation with 5 μl Strataclean resin (Agilent Technologies). Resin was pelleted by centrifugation, resuspended in 2X sample loading buffer then boiled for 5 min in preparation for 12% SDS-PAGE/Western immunoblot analysis using anti-GSK-3β (Cell Signalling Technology Inc), anti-GAPDH or anti-CagA (Santa-Cruz Biotechnology) antibodies.

### Inhibitor studies

Chemical inhibitors 6 amino-4-(4-phenoxyphenylethylamino) quinazoline (1 μM; NF-κB activation inhibitor, Merck-Millipore), U0126 (10μM; MEK1 inhibitor) and SP600125 (10μM; c-Jun-N-terminal kinase [JNK] inhibitor) were added to epithelial cell monolayers 1 h before and throughout the infection period. Control cells were treated with an equivalent volume of DMSO (inhibitor solvent) alone in the same manner both in the presence and absence of *H*. *pylori* infection.

### Statistical analysis

Statistical analyses were performed using GraphPad Prism 6.00 (GraphPad Software, California, USA). Means and SDs were used to describe *in vitro* data and subsequently analysed using Student’s *t*-test or one-way analysis of variance with Dunnett’s post hoc test for multiple comparisons as appropriate. A *p* value ≤0.05 was taken to denote statistical significance.

## Results

### CtkA is variably encoded by the *tfs3* ICE

In a previous PCR-based survey, the prevalence of the *ctkA* gene was found to vary widely in different geographic populations, ranging from 1.5% (Brazil) to 100% (South Africa) [13]. Significant population and regional variation is further suggested here by protein and nucleotide BLASTP/N searches for *ctkA* sequence in the >500 *H*. *pylori* genomes in GenBank, currently identifying a total of only 30 sequences, predominantly in strains originating from Asian (Malaysia, Japan and India) and South American (Colombia and Peru) countries. Sequence conservation between all homologues is high (93–99% identity over the entire sequence) and similarly observed between the *H*. *pylori ctkA* and a homologous gene present in two genome sequenced strains of *H*. *cetorum (*MIT 99–5656 and MIT 00–7128).

**Fig 1:**
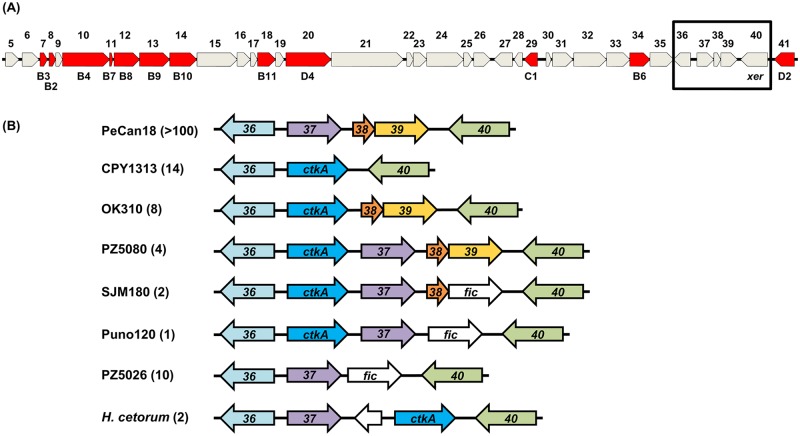
Genetic context of *ctkA* in *H*. *pylori tfs3* ICE gene clusters. (A) The *tfs3* ICE of *H*. *pylori* strain PeCan18b. Genes are annotated according to the ‘*pz*’ gene designation (*pz1-41*) initially used to describe the *tfs3* gene cluster of strain PeCan18b. Corresponding genes in selected reference genomes are provided in S3 Table. *vir*-homologous genes (*vir*B2-11 [T4SS assembly], *vir*D4, *vir*D2 [relaxase], *vir*C1 [ancillary relaxosome protein]), encoding key components associated with the putative Tfs3 T4SS are coloured red and labelled accordingly. (B) The variable context of *ctkA* within comparable ends of the *tfs3* ICE gene cluster is shown for representative strains with reference to a strain lacking *ctkA* (PeCan18). Additional *ctkA*+ strain genomes in GenBank with an equivalent organisation of genes to those shown are indicated in parentheses after the named reference strains and are as follows; CPY1313 (CPY6261, UM038, UM066, UM085, UM370, UM411, FD577, FD703, A-27, L7, 83, 22, J99), OK310 (UM303R/S, BM013A/B, FD506, ML3, 59, F32, UM096), PZ5080 (NQ4076, NQ4099, NQ4200) and SJM180 (Puno135), Puno120, PZ5026 (H-1, H-16, H-18, H-36, P-2, P-26, Aklavik117, Shi112, PeCan4), *H*. *cetorum* (strains MIT 00–7128 and MIT 99–5656). NCBI GenBank Accession numbers for strain sequences shown are; PeCan18 (AF487344 and CP00347), CPY1313 (AKNK01), OK310 (AP012601), PZ5080 (ASYV01), SJM180 (CP002073), Puno120 (CP002980), PZ5026 (ASYT01) and *Helicobacter cetorum* MIT 00–7128 (CP003479).

Further examination of the genetic context of *ctkA* shows it to be located close to *xer* and *virD2* genes towards the end of the *tfs3* ICE in *H*. *pylori* and both *H*. *cetorum* genomes (Fig 1A). However, although consistently located immediately 5’ proximal to the *pz36* gene in *tfs3* (as assigned in the original annotation of *tfs3* from strain PeCan18b, accession number AF487344), the genetic context downstream of *ctkA* appears more variable; although occurring in addition to the existing complement of *tfs3* genes in several strains, most often, the presence of *ctkA* is coincident with the loss of either *pz37* or *pz37-39*.

In *tfs3* types where *pz37-39* are absent, *ctkA* is arranged in reverse orientation with respect to the *tfs3 xer* gene, in a position more frequently occupied by homologues of the *pz39* gene or more rarely, a gene with homology to the Fic superfamily of encoded proteins (Accession pfma02661, E-value 7.39e-06). This latter includes cell division proteins and components of addiction (toxin/antitoxin) modules involved in prophage/plasmid stability. Whereas *ctkA* and *fic* occur rarely in the genomes sequenced to date, the *pz39* gene is relatively abundant by comparison (> 100 sequences identified in GenBank by BLASTP) and of the three, seems to be most commonly associated with the *tfs3* cluster. Considering this variation, the *tfs3* ICE can be broadly classified by *ctkA*, *fic and pz39* presence/absence and genetic disposition into the eight variant types illustrated in Fig 1B.

**Fig 2:**
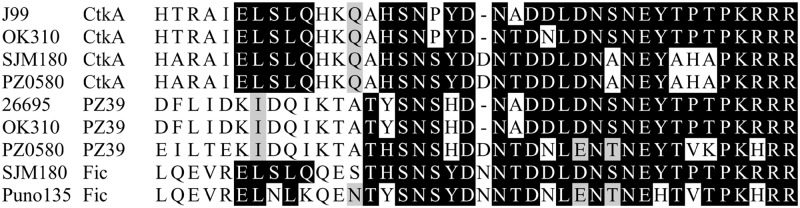
Sequence alignments showing conservation of C-terminal sequence between *tfs3* ICE-encoded proteins. Sequence alignment demonstrates conservation in the last 25 C-terminal amino acid residues of CtkA, PZ39 and Fic proteins encoded in *tfs3* gene clusters from a representative selection of *H*. *pylori* strains.

Although not obviously related, CtkA, PZ39 and Fic notably all share a conserved stretch of 27 residues at the extreme C-terminus of each protein (Fig 2), over which CtkA is 66.7% and 77.8% identical to the equivalent region of PZ39 and Fic respectively. BLASTP of CtkA sequence also identifies Ser/Thr kinase homologues in many other gastrointestinal organisms, including *Helicobacter bizzeronii*, *Enterococcus faecium* and *Clostridium sp*. which share sequence similarity over the entire length of the protein (~50% identity) except for the extreme C-terminal sequence, which is either absent or sequence divergent. This suggests that the C-terminal tail is an acquired feature peculiar to these *H*. *pylori* proteins, and by virtue of its sequence conservation, may present a downstream recombination site for generation of some of the observed variation in *tfs3* gene content.

### CtkA expressed from *tfs3*+ *H*. *pylori* strains induces pro-inflammatory cytokine secretion independently of the Cag type IV secretion system

Given the association of *ctkA* with the *tfs3* ICE (Fig 1), and the evident ability of rCtkA to interact with host immune cells [12, 13], we considered that CtkA might require T4SS components of the *tfs3* ICE for its release from *H*. *pylori*. As a first step to investigate this, a strategy was developed to enable expression and facile detection of CtkA from *tfs3*+/*cag* T4SS–*H*. *pylori* strain backgrounds. A requirement for Cag T4SS mutant strains was considered important since the *cag*PAI-encoded T4SS is well known to stimulate a potent pro-inflammatory cytokine response [31–33] which would likely confound assessment of the substantially more modest pro-inflammatory responses previously reported as a consequence of rCtkA interaction [12, 13].

Accordingly, we first examined a selection of *H*. *pylori* strains for the presence of *tfs3* by PCR typing of eight *tfs3* genes located at intervals along the length of the *ca*. 40kbp *tfs3* ICE. Three strains (AB31, AB5, 10A) typed positive for all eight markers suggesting the presence of complete *tfs3* gene clusters and one strain was found to lack *tfs3* altogether (64) (Table 1). These strains were additionally typed for markers of the *tfs4* ICE and *cag*PAI-encoded T4SSs. *ctkA* could not be detected in any strain background except for the PCR control strain J99, which is known to contain an extensively rearranged and fragmented mosaic *tfs3/4* gene cluster [27, 28].

**Table 1:** PCR typing of *H*. *pylori* strains.

Strain	*tfs3 pz gene*								Other *tfs/cag* genes				
	*pz5*	*pz9*	*pz12*	*pz20*	*pz26*	*pz31*	*pz35*	*pz40*	*ctkA*	*dupA*^1^	*virB9*^1^	*cagE*	*cagA*
AB31	+	+	+	+	+	+	+	+	-	+	+	+	+
AB5	+	+	+	+	+	+	+	+	-	-	-	+	+
10A	+	+	+	+	+	+	+	+	-	-	-	+	+
64	-	-	-	-	-	-	-	-	-	+	+	+	+
J99	-	-	-	-	+	-	+	-	+	+	+	+	+

^1^component genes of the tfs4 ICE

Next, we generated a construct comprising the *H*. *pylori flaA* promotor fused to a 5’-*gsk*-tagged *ctkA* sequence amplified from strain J99, cloned adjacent to a kanamycin resistance cassette and flanked by 5’ and 3’ ends of *cagE* in the pGEMT-Easy cloning vector. Subsequent transformation of the *tfs3*-typed *H*. *pylori* (*cagE*+) strains with the pGEM*cagE*::*gsk-ctkA-kan* plasmid resulted in efficient insertional inactivation of *cagE* with the *ctkA* expression cassette in each, enabling constitutive *in cis* expression of *ctkA* in single copy with concomitant inactivation of the Cag T4SS. An equivalent set of *cagE* deletions were also constructed more conventionally by insertional inactivation with the kanamycin resistance cassette alone for use in control studies.

**Fig 3:**
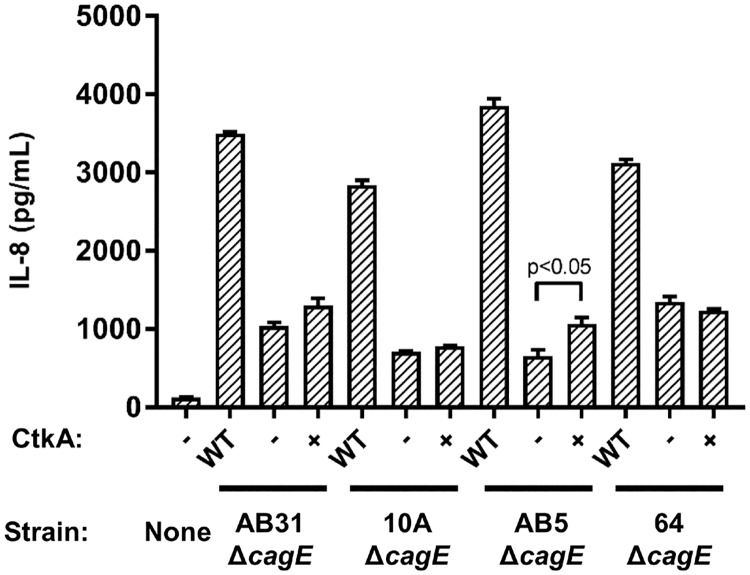
CtkA expressed from *H*. *pylori* stimulates pro-inflammatory cytokine secretion from gastric epithelial cells. Wild-type *H*. *pylori*, *cagE-*deficient strains and isogenic derivatives constitutively expressing GSK-CtkA were assessed for their ability to induce IL-8 secretion from MKN28 cells in co-culture. Supernatants were sampled 48 h post infection and assayed for IL-8 secretion. In comparison with the parent *cagE* mutants alone, all *tfs3*(+) strains expressing GSK-CtkA (AB31, 10A, AB5) showed a trend towards induction of elevated levels of IL-8 when expressing GSK-CtkA, whereas a *tfs3*(-) strain (64) did not. The difference in levels of induced IL-8 due to GSK-CtkA expression was significant when expressed from strain AB5. Levels of IL-8 attributable to GSK-CtkA were modest relative to *cag*PAI-mediated responses from all wild-type strains. Graph shows means and SDs from three independent experiments, each performed in triplicate.

Each of the four GSK-CtkA expressing *cagE* mutant strains, AB31Δ*cagE*::*gsk-ctkA-kan*, AB5Δ*cagE*::*gsk-ctkA-kan*, 10AΔ*cagE*::*gsk-ctkA-kan* and 64Δ*cagE*::*gsk-ctkA-kan* were then incubated in co-culture with MKN28 gastric epithelial cell monolayers for 48 h, in parallel with their respective parent strain or *cagE* isogenic mutants. Subsequent assay of supernatants for IL-8 cytokine secretion showed a trend towards increased levels of IL-8 in response to co-culture with the CtkA-expressing strains compared to the *cagE* mutants alone in all three *tfs3*+ strains (Fig 3). However, the difference only reached significance with the AB5 strain pair. IL-8 levels were not observed to increase in the *tfs3*- strain 64Δ*cagE*::*gsk-ctkA-kan* despite broadly equivalent expression of GSK-CtkA.

Interestingly, the level of IL-8 secreted in response to infection of gastric epithelial cells with AB5Δ*cagE*::*gsk-ctkA-kan* was broadly comparable with levels previously observed from stimulation of immune cells using purified rCtkA [12, 13]. Notably, however, these levels were modest compared with the potent stimulation attributable to the Cag T4SS, which in this study were found to be ~6x higher following infection with the AB5 wildtype (*cag+*) strain (mean IL-8 concentrations of 3853 pg/mL vs 658 pg/mL for wildtype and *cagE* mutant respectively). These results indicate that CtkA, when expressed from *H*. *pylori*, can stimulate pro-inflammatory cytokine secretion from gastric epithelial cells and that CtkA-mediated induction of IL-8 secretion, although modest, occurs independently of either the Cag or putative Tfs4 T4SS.

### CtkA expressed from *H*. *pylori* induces IL-8 secretion by activation of NF-κB

Previous studies using both recombinant and transfected CtkA established that induction of cytokine secretion in response to CtkA stimulation is mediated by NF-κB signalling [12]. Since both physical/biochemical properties of CtkA and the manner in which it is presented to a host cell may differ following expression and presumably secretion from a native *H*. *pylori* host compared with either transfected or rCtkA expressed and purified from a heterologous host (*Escherichia coli*), it was important to confirm that the underlying mechanism of host-cell stimulation by both native and recombinant proteins was analogous.

To investigate this, the MKN28 gastric epithelial cell co-culture model was again employed to examine the effect of selective inhibition of signalling pathways in the CtkA-mediated secretion of IL-8. The AB5 strain background was used for these experiments since we had observed a greater effect of GSK-CtkA-mediated induction of IL-8 secretion from this strain compared with other strains (Fig 3) and advantageously, AB5 appeared to also lack components of the Tfs4 T4SS (Table 1), enabling any possible contribution from this related system to be discounted.

**Fig 4:**
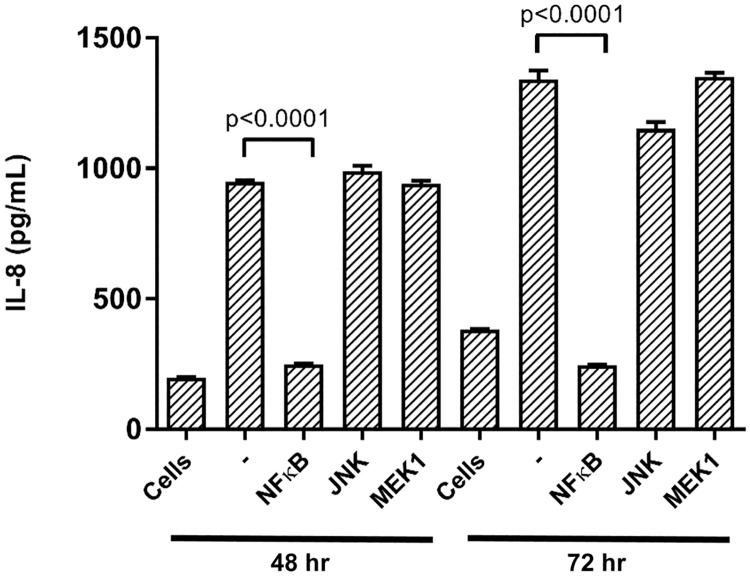
Signalling pathways involved in CtkA-mediated stimulation of IL-8 secretion. MKN28 cell monolayers remained untreated (-), or were treated with chemical inhibitors for NF-κB (6 amino-4-(4-phenoxyphenylethylamino) quinazoline), JNK (SP600125) and MEK1 (U0126) for 1 h before and during infection with *H*. *pylori* strain AB5Δ*cagE*::*gsk*-*ctkA*. IL-8 levels were subsequently assessed by ELISA. Basal levels of IL-8 detected in uninfected supernatants are also indicated for reference (‘Cells’). Graph shows means and SDs from three independent experiments, each performed in triplicate. *p* values indicate significant differences in the presence of inhibitor compared with infection alone.

Parallel MKN28 co-cultures with the AB5Δ*cagE*::*gsk-ctkA-kan* strain were incubated in the presence or absence of specific pharmacologic inhibitors of NF-κB activation and of the MAP kinases MEK1 and JNK. Assessment of IL-8 levels in the supernatants of infected monolayers showed that NF-κB inhibition significantly reduced induction of IL-8 secretion in response to AB5Δ*cagE*::*gsk-ctkA-kan* infection, essentially to baseline levels (Fig 4). Conversely, inhibitors of JNK and MEK1 had negligible effects, indicating that pathways involving these kinases, including Toll-like receptor signalling, are not significantly involved in CtkA-mediated IL-8 cytokine responses. These results therefore indicate that *H*. *pylori* expressed CtkA engages with host cells to induce pro-inflammatory signalling in a similar manner as rCtkA.

### CtkA interaction with gastric epithelial MKN28 cells requires the Tfs3 T4SS

To more formally investigate the role of the putative Tfs3 T4SS in CtkA-mediated pro-inflammatory signalling, we targeted the *tfs3*-encoded *virB9* homologous gene in strain AB5 for insertional inactivation with a chloramphenicol resistance cassette. VirB9 is an essential structural component required for substrate selection and assembly of the T4SS complex [34]. Close examination of the genetic context of the *virB9* gene in the genome sequence of strain PeCan18 indicates it to be located centrally within a large putative operon spanning at least genes *pz5-pz21* (Fig 1A). As insertional inactivation of *virB9* likely evokes polar effects on downstream genes, including others also required for T4SS assembly/activity (*virB10*, *virB11*, *virD4*) the resulting mutant, termed AB5Δ*virB9*Δ*cagE*::*gsk-ctkA* for brevity, was confidently considered to be entirely abrogated in T4SS function.

**Fig 5:**
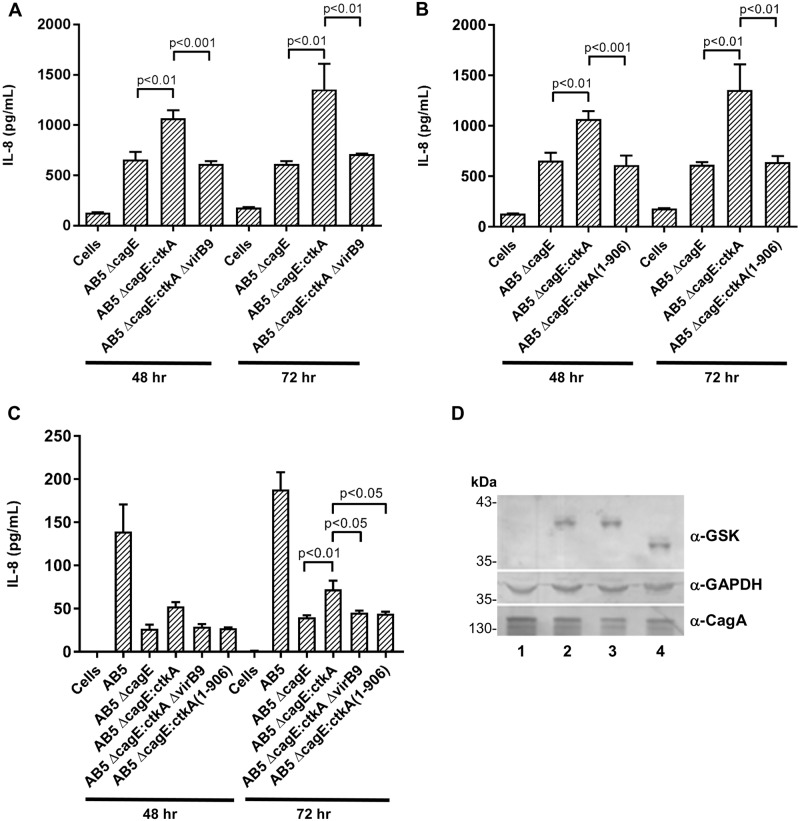
Pro-inflammatory signalling in response to CtkA requires the Tfs3 type IV secretion system and the C-terminus of CtkA. MKN28 cells were co-cultured with *H*. *pylori* AB5Δ*cagE* derivative strains (MOI = 20) for 48 or 72 h prior to determination of IL-8 concentrations in supernatants by ELISA. Both inactivation of (A) *tfs3 virB9* and (B) deletion of the last 23 C-terminal amino acid residues of CtkA in strain AB5Δ*cagE*::gsk-*ctkA*(1–906) resulted in abrogation of IL-8 secretion. (C) Similar effects were observed following co-culture of the complement of AB5 strains with gastric AGS cells although IL-8 responses for all strains were markedly lower. Graph shows means and SDs from three independent experiments, each performed in triplicate. *p* values indicate significant differences in IL-8 levels compared to the AB5Δ*cagE*::*gsk-ctkA* strain. (D) Western immunoblot analysis of 100X concentrated supernatants from *H*. *pylori* strains AB5Δ*cagE*, AB5Δ*cagE*:*gsk*-*ctkA*, AB5Δ*virB9*Δ*cagE*:*gsk*-*ctkA* and AB5Δ*cagE*:*gsk*-*ctkA*_1-906_ (Lanes 1–4 respectively) grown in RPMI 1640. Blots were probed with anti-GSK, anti-GAPDH and anti-CagA specific antiserum. Detection of GAPDH and CagA in supernatants indicates non-secretory release of intracellular protein in all strain backgrounds.

Notably, assay of supernatants following co-culture of AB5Δ*virB9*Δ*cagE*::*gsk-ctkA* with MKN28 cell monolayers as before determined that VirB9/Tfs3 T4SS mutation totally abrogated induction of IL-8 secretion by the AB5Δ*virB9*Δ*cagE*::*gsk-ctkA* strain (Fig 5A), providing the first indication for involvement of the Tfs3 T4SS in pro-inflammatory signalling by CtkA.

Cell monolayers from these experiments were also assessed for the presence of intracellular/translocated CtkA by immunoblot of lysates with phospho-GSK antibodies as reported previously for CagA (Hohlfeld *et al*., 2006). However, despite evident host cell interaction by CtkA, host-specific phosphorylation of the GSK tag could not be obviously detected in epithelial cell monolayers infected with the AB5Δ*cagE*::*gsk-ctkA-kan* strain, possibly due to low levels of intracellular CtkA protein.

In view of this, we focused on defining other correlates of CtkA export from *H*. *pylori* and next considered properties of T4SS secretion substrates that enable their recognition by a cognate T4SS. Many T4SS secreted proteins, including the majority of *Agrobacterium tumefaciens* T4SS substrates comprise a C-terminal secretion signal characterised by clusters of positively charged residues which are considered to mediate electrostatic contacts with the coupling protein/VirD4 component of the T4SS [35–37]. Consistently, CtkA sequence also comprises an equivalent charged cluster (KRRR) in the last four C-terminal residues of the protein similarly shared with both PZ39 and Fic (Fig 2). Therefore, accounting for any possible requirement for broader sequence context, we next generated a mutant CtkA lacking the last 23 C-terminal residues and introduced this into *cagE* of strain AB5 for *in cis* expression as before. Subsequent assay of IL-8 secretion from MKN28 monolayers infected with strain AB5Δ*cagE*::*gsk-ctkA*_*1-906*_ indicated a similar abrogation of IL-8 levels as we observed for the Tfs3 T4SS mutant AB5Δ*virB9*Δ*cagE*::*gsk-ctkA* strain (Fig 5B), demonstrating the importance of the C-terminal tail for CtkA activity in the context of *H*. *pylori* infection. Co-culture of the complement of AB5 wild-type and mutant strains with gastric AGS cells showed similar effects for each strain (Fig 5C) although IL-8 levels for all strains were lower compared with levels observed from MKN28 cells.

Since previous studies had reported dose-dependent induction of both IL-8 and TNF-α secretion from cultured macrophage and mononuclear cells in response to treatment with rCtkA [12, 13] co-culture supernatants were also assayed for the presence of TNF-α. However, in contrast to observations with immune cells, TNF-α could not be detected in supernatants following co-culture of any strain with either MKN28 or AGS cell lines within the detectable limits of the assay (>3 pg/mL).

Finally, to investigate CtkA release independently of the presence of host cells, the complement of AB5Δ*cagE*::*gsk-ctkA* strain backgrounds were incubated in RPMI 1640 for 24 h to simulate co-culture growth conditions, then 100X concentrated culture supernatants examined for the presence of GSK-CtkA proteins using anti-GSK antibodies. Surprisingly, GSK-CtkA was apparent in the supernatants of all strains at broadly equivalent levels (Fig 5D, top panel). However, further examination also revealed the presence of both GAPDH and CagA, similarly at roughly equivalent levels in all supernatant samples (Fig 5D, middle and bottom panels). As secretion of CagA is dependent upon the Cag T4SS and host cell contact [6, 7, 38] we conclude that its presence in supernatants, and that of cytosolic GAPDH, is indicative of non-secretory release, due perhaps to either presence of outer membrane vesicles, or a tendency of strain AB5 towards lysis in liquid culture. However, although precluding direct examination of host-cell dependent stimulation of CtkA secretion in the AB5 background, it remains notable that the level of CtkA protein present in supernatants is not sufficient to appreciably stimulate IL-8 secretion from epithelial cells (Fig 5A and 5B). Therefore, supernatant-free CtkA in this assay does not appear to account for the cytokine response observed from co-culture with AB5Δ*cagE*::*gsk-ctkA*.

Collectively, these results indicate that both *tfs3* ICE-encoded T4SS assembly proteins and C-terminal CtkA sequence may be specifically required for delivery of CtkA to host cells for subsequent interaction and stimulation of CtkA-mediated responses.

## Discussion

The *tfs3* and *tfs4* gene clusters are considered to be self-transmissible integrative and conjugative elements which are variably distributed in diverse *H*. *pylori* strains. Although both *tfs* ICEs comprise a complement of *vir*-homologous genes encoding likely components of a T4SS, the role of these systems and identification of the proteins they secrete has not been determined. Here, we provide the first evidence of a role for the putative Tfs3 T4SS in promoting host cell interaction of the pro-inflammatory protein CtkA.

The *ctkA* gene has been identified in several studies as a potential marker of gastroduodenal disease and the encoded product, expressed and purified as recombinant protein from a heterologous host, shown to induce pro-inflammatory cytokine secretion from human macrophage and monocyte cell lines [12, 13]. In this study, we show that CtkA expressed from its native *H*. *pylori* host has similar activity as rCtkA, providing important perspective for these previous observations in the context of *H*. *pylori* infection. Moreover, this work determines that CtkA activity is similarly directed to gastric epithelial cells and further indicates that native-host expressed CtkA has no apparent species-specific requirement for post-translational modification or assisted structural folding, either through the contribution of a species or T4SS-specific chaperone or the mechanism of CtkA export itself.

Of particular consequence, we demonstrate that induction of CtkA-dependent IL-8 secretion is almost entirely abrogated in the Tfs3 T4SS mutant strain AB5Δ*virB9*Δ*cagE*::*gsk-ctkA* (Fig 5A). This observation provides the first indication of a functional role for the Tfs3 T4SS as a virulence-associated secretion system and identifies host-interacting CtkA as a putative Tfs3 T4SS effector substrate.

This idea is further supported by the finding that deletion of the C-terminus of CtkA also abolishes the IL-8 phenotype (Fig 5B). Previous work with rCtkA reported that the C-terminal tail of the protein (residues 301–325) was not required for either the ability of rCtkA to translocate across the host plasma membrane or for the subsequent enhancement of NF-κB activity or cytokine secretion [12]. Consequently, the lack of stimulation due to CtkA C-terminal truncation observed in our infection model likely reflects reduced availability of CtkA, which in the context of our collective results, we consider to be consistent with a CtkA-secretion defect.

Our inability to detect host-mediated phosphorylation of the CtkA GSK-tag in infected monolayers, in spite of highly reproducible, albeit modest, pro-inflammatory stimulation by CtkA, was initially puzzling. However, the limited pro-inflammatory responses that we observe may be indicative of a general low abundance of intracellular CtkA, possibly due to an unknown deficiency in our experimental model or as a reflection of actual, physiologically low levels of CtkA which relate to the role of CtkA in *H*. *pylori* infection.

Paradoxically, whereas non-secretory CtkA is present at various levels in the supernatants of all strains examined, the cell-free released protein appears insufficient to stimulate epithelial cells in co-culture (Fig 5). This is in apparent contrast to observations with rCtkA [12, 13]. However, these latter studies observed cytokine effects from co-culture with PBMCs and differentiated Thp1 macrophage-like cells rather than gastric epithelial cells, and effects were most readily detected using concentrations of rCtkA upwards of 0.5μg ml^-1^ of protein, which is likely to be far in excess of the levels of native protein released into supernatants from *H*. *pylori* in our experiments. Regardless, that CtkA free in the supernatant does not significantly stimulate IL-8 secretion from MKN28 cells in co-culture further emphasises a requirement for the Tfs3 T4SS and importantly suggests that CtkA secretion occurs closely proximal to the host cell rather than freely into the surrounding medium. Presumably, the levels of IL-8 attributable to CtkA we observe in the presence of *H*. *pylori* reflects a dose-dependent delivery of CtkA, although whether this is achieved by CtkA export to the surface of either the attached bacterial or host cell, or directly to the host cytoplasm is presently undetermined. However, as previous studies have clearly indicated an ability of rCtkA to self-translocate across the host plasma membrane [12], it seems reasonable that Tfs3 T4SSmediated delivery of CtkA to the host cell surface would be sufficient for promotion of subsequent CtkA activities.

We have shown in this study that the C-terminal sequence of the CtkA protein potentially comprises a signal for T4SS-mediated secretion. T4SS secretion signals are invariably located at the extreme C-terminus of the secretion substrate. This is the case for the *H*. *pylori* CagA protein [39] and numerous secretion substrates of the archetypal *A*. *tumefaciens* Vir T4SS [37]. Whereas the secretion signal remains largely indistinct, the C-terminal sequence of all these proteins tend to share a charge bias of positive residues, commonly require a hydrophobic or proline residue in the -3 or -4 position relative to the terminal amino acid, and are predicted to lack structure [35–37, 40, 41]. These features are considered to facilitate solvent accessibility of the C-terminus, enabling its productive interaction with the VirD4 type IV coupling protein (T4CP) receptor via charged amino acid contacts [37]. Notably, these features are all evident in the conserved C-terminal sequence of CtkA, which displays both a bias of positively-charged amino acids (KRRR) and a proline residue, albeit in the -5 position (Fig 2). Furthermore, the crystal structure of CtkA is C-terminally truncated due to lack of electron density in the last 25 residues, consistent with secondary structure prediction indicating that this region of CtkA is intrinsically disordered. As the characteristic C-terminal signal sequence is absent from homologous Ser/Thr kinases from other gastrointestinal bacteria, we speculate that it may be an acquired feature that confers secretion competence, thereby marking CtkA for recognition as a T4SS secretion substrate. Other *tfs3*-encoded proteins PZ39 and Fic which also share conserved C-terminal sequence (Fig 2) may similarly be highlighted as candidate secretion substrates, although a role for these proteins in host interaction and virulence has yet to be explored.

The repertoire of proteins secreted by a particular pathogen significantly contributes to the nature and outcome of its interaction with a host. Many studies indicate that *H*. *pylori* strains encoding the VirB4 (i.e. DupA) component of the Tfs4 T4SS, and most likely the Tfs4 T4SS itself, are more strongly associated with a risk of duodenal ulcer disease [25]. However, no equivalent studies to our awareness have overtly reported the disease association of *tfs3 virB4* or any other (*vir* gene) marker of the Tfs3 T4SS, although several have reported an association of *ctkA* with increased or decreased risk of gastroduodenal disease in some populations [14, 16, 18, 19]. Of these, the study of Occhialini *et al* [14] indicated an association of *ctkA* (*jhp0940*) with strains isolated from gastric cancer patients, where 42% of strains encoded *jhp0940* compared with none of the gastritis isolates. Advantageously, their data also included the distribution of several other *tfs3* genes including *jhp0937* encoding the putative Tfs3 VirB6 protein (S3 Table), which, as a likely component required for T4SS assembly can be considered a marker for the Tfs3 T4SS. Re-examination of the data finds *jhp0937* present in 70.5% of gastric cancer isolates compared with 48% of gastritis isolates and significantly, all but one of the isolates which also encoded CtkA. There is therefore some suggestion that the presence of the Tfs3 T4SS also correlates with an increased risk for gastroduodenal disease in certain populations, and certainly those in which CtkA appears important for disease risk. In view of this and our collective findings, it would be relevant to examine the distribution of a broader subset of *tfs3* genes in different patient populations to more definitively assess the importance of the Tfs3 T4SS and the proteins it secretes in defining the virulence potential of particular *H*. *pylori* strains.

## Supporting information

S1 Table Plasmids and strains. (DOCX)

S2 Table Oligonucleotides. (DOCX)

S3 Table *tfs3 pz* gene homologues. (DOCX)
