# Supplementary material for: A role for the tfs3 ICE-encoded type IV secretion system in pro-inflammatory signalling by the Helicobacter pylori Ser/Thr kinase, CtkA
Source: PLoS One. 2017 Jul 28;12(7):e0182144. doi: 10.1371/journal.pone.0182144 (PMC5536186; doi:10.1371/journal.pone.0182144)
Supplement: S1 Table — (DOCX) [file pone.0182144.s001.docx]

**S1 Table.** Plasmids and strains

| **Plasmid or strain** | **Description** | **Reference or source** |
| --- | --- | --- |
| **Plasmids:** |  |  |
| pGEM-TEasy | High copy number cloning vector, Ap^R^ | Promega |
| pSB14 | *E. coli-H. pylori* shuttle vector for expression of inserts under the control of the *H. pylori flaA* promotor (*fla*^P^), Km^R^ | [^42^](#_ENREF_42) |
| pSB14-*gsk-ctkA* | pSB14 expressing GSK-tagged CtkA | This study |
| pGEM-*cagE::kan* | pGEM-TEasy containing a kanamycin resistance cassette inserted within *cagE* coding sequence | This study |
| pGEM-*cagE*::*gsk-ctkA-kan* | pGEM-TEasy containing *fla*^P^-*gsk-ctkA*-*kan* inserted within *cagE* coding sequence | This study |
| pGEM-*cagE*::*gsk-ctkA_1-906_-kan* | As pGEM-*cagE*::*gsk-ctkA-kan* but encoding CtkA with a truncation of the last 23 amino acid residues (ΔC23) | This study |
| pGEM-*virB9::CAT* | pGEM-TEasy containing a kanamycin resistance cassette inserted within *tfs3 virB9* coding sequence | This study |
| **Strains:** |  |  |
| *E. coli* XLI-Blue | *recA1 endA1 gyrA96 thi-1 hsdR17 supE44 relA1 lac* [F´ *proAB lacI*q*ZΔM15* Tn*10* (Tetr)]. | Agilent Technologies |
| J99 | Reference *H. pylori* strain | [^43^](#_ENREF_43) |
| 26695 | Reference *H. pylori* strain | [^44^](#_ENREF_44) |
| AB31 | Clinical *H. pylori* strain | [^45^](#_ENREF_45) |
| AB5 | Clinical *H. pylori* strain | [^45^](#_ENREF_45) |
| 10A | Clinical *H. pylori* strain | [^46^](#_ENREF_46) |
| 64 | Clinical *H. pylori* strain | [^30^](#_ENREF_30) |
| AB31Δ*cagE* | *cagE* deletion | This study |
| AB5Δ*cagE* | *cagE* deletion | This study |
| 10AΔ*cagE* | *cagE* deletion | This study |
| 64Δ*cagE* | *cagE* deletion | This study |
| AB31Δ*cagE*::*gsk-ctkA-kan* | *cagE* deletion strain expressing GSK-CtkA *in cis* | This study |
| AB5Δ*cagE*::*gsk-ctkA-kan* | *cagE* deletion strain expressing GSK-CtkA *in cis* | This study |
| 10AΔ*cagE*::*gsk-ctkA-kan* | *cagE* deletion strain expressing GSK-CtkA *in cis* | This study |
| 64Δ*cagE*::*gsk-ctkA-kan* | *cagE* deletion strain expressing GSK-CtkA *in cis* | This study |
| AB5Δ*cagE*:: *gsk-ctkA_1-906_-kan* | *cagE* mutant strain expressing GSK-CtkA(ΔC23) *in cis* | This study |
| AB5Δ*virB9*Δ*cagE*::*gsk-ctkA-kan* | *cagE* and *tfs3 virB9* double mutant strain expressing GSK-CtkA *in cis* | This study |
