# Supplementary material for: A role for the tfs3 ICE-encoded type IV secretion system in pro-inflammatory signalling by the Helicobacter pylori Ser/Thr kinase, CtkA
Source: PLoS One. 2017 Jul 28;12(7):e0182144. doi: 10.1371/journal.pone.0182144 (PMC5536186; doi:10.1371/journal.pone.0182144)
Supplement: S2 Table — (DOCX) [file pone.0182144.s002.docx]

**S2 Table.** Oligonucleotides

| **Name** | **Oligonucleotide sequence (5’ – 3’)** |
| --- | --- |
| TP-pz5F | GATGAGTTAAACACACTTTATAAGGC |
| TP-pz5R | TCTTCACAATGGCTGTTTGATAG |
| TP-pz9F | CAAAAAGAAGTCTTAGTAGAAAAACC |
| TP-pz9R | TTAAGAAGACTTTCTTTTTCTTTTAGG |
| TP-pz12F | TGACAAAATCCGCTTGAATG |
| TP-pz12R | TGCTTGCGATTTCACTCAAG |
| TP-pz20F | AAAATGCAATTTGTGAAGGC |
| TP-pz20R | CAATGTTATAATGCACCACGC |
| TP-pz26F | AAGCGAACAAGCTCTTAATCATG |
| TP-pz26R | TATCGCCTACGCTTAGGTGTG |
| TP-pz31F | AAAGCGTGGAATGAAATGG |
| TP-pz31R | GCTTGGATAAATCATAAATCAC |
| TP-pz35F | GATACAACTATGAGAATTTAAAGCAA |
| TP-pz35R | TGATTTAAAAAGAGCATTTGATT |
| TP-pz40F | GATGCAAGGCTTGTTTTTATTC |
| TP-pz40R | GATGTAAGCCTGTGCCATATTG |
| TP-ctkAF | TCAAGAAACATTGCTAGGCACT |
| TP-ctkAR | TGATTTTCTTTCATCAAGCATTG |
| TP-dupAF | TTCACGCCTAAGACCTCAAAC |
| TP-dupAR | TGAGAAGCCTTATTATCTTGTTGG |
| TP-tfs4virB9F | TTCTTTTGAACATGGCAAAGA |
| TP-tfs4virB9R | TAGGTTGAGGCGTAGTGTCTTC |
| TP-cagEF | TGATTGTAGCGATTGTTATTGTG |
| TP-cagER | TGACATACTCCCCACCCATT |
| cagEiPXmR | CTATATCCCGGGAACTTCTTTCACCCCACCAT |
| cagEiPXhF | CAATACCTCGAGGAATACGCCCCTAAATCACA |
| kanXmBF | CAATACCCCGGGATAGGATCCTCATGTTTGACAGCTTATCATCG |
| kanXhR | GGACTGCTCGAGAATCTAGGTACTAAAACAATTCATCC |
| SBflaAXmF | CAATACCCCGGGCCTTTGAGTGAGCTGATACG |
| apha3R | CGGGGAAGAACAGTATGTCG |
| cagEvalidF | ATAGAGCAAGAGGTTCAAAAGC |
| cagEvalidR | GGCAAGCCAAAAGATCAGTGATG |
| tfs3virB9F | ATTCAAAATTATAATGAGCCTATGG |
| tfs3virB9R | TCTTGCCCTCTTTGCTATCTT |
| tfs3B9iPXmR | CTATATCCCGGGCCAAGCATTTTAGTAGAAAAGCC |
| tfs3B9iPXhF | CAATACCCCGGGATACTCGAGCATTGGAAAAAGCGGTAAGG |
| GSK-ctkANdF | GAACTGCATATGAGTGGTCGCCCTCGCACTACTAGTTTCGCTGAAAGTCTCGAGCCAACCATTGATTTTAC |
| ctkASR | ATTCGGGTCGACTTATCGTCTACGCTTAGGTGTG |
| ctkASR2 | ATTCGGGTCGACTTATGAGTGAGCTTGTTTGTGTTGTA |
| ctkABR2 | ATTCGGGGATCCTTATGAGTGAGCTTGTTTGTGTTGTA |
