# Supplementary material for: A role for the tfs3 ICE-encoded type IV secretion system in pro-inflammatory signalling by the Helicobacter pylori Ser/Thr kinase, CtkA
Source: PLoS One. 2017 Jul 28;12(7):e0182144. doi: 10.1371/journal.pone.0182144 (PMC5536186; doi:10.1371/journal.pone.0182144)
Supplement: S3 Table — (DOCX) [file pone.0182144.s003.docx]

**S3 Table.** *tfs3 pz* gene homologues

|  |  | ***H. pylori* strain** | | |
| --- | --- | --- | --- | --- |
| ***tfs3 pz* gene** | ***vir* gene homologue** | **PeCan18**  (HPPC18_) | **P12**  (HPP12_) | **J99**  (*jhp*_) |
| 5 | - | 5050 | 1320 | - |
| 6 | *-* | 5040 | 1322 | - |
| 7 | *virB2* | 5025 | 1323 | - |
| 8 | *virB3* | 5020 | 1324 | - |
| 9 | *-* | 5015 | 1325 | - |
| 10 | *virB4* | 5010 | 1326 | - |
| 11 | *virB7* | 5005 | 1327 | - |
| 12 | *virB8* | 5000 | 1328 | - |
| 13 | *virB9* | 4995 | 1329 | - |
| 14 | *virB10* | 4990 | 1330 | - |
| 15 | *-* | 4985 | 1331/1332 | - |
| 16 | *-* | 4980 | 1333 | - |
| 17 | *-* | 4975 | 1334 | - |
| 18 | *virB11* | 4970 | 1335 | - |
| 19 | *-* | 4965 | 1336 | - |
| 20 | *virD4* | 4960 | 1337 | - |
| 21 | *-* | 4955 | - | - |
| 22 | *-* | 4950 | - | - |
| 23 | *-* | 4940 | - | 0930 |
| 24 | (*topA*) | 4935 | 1338 | 0931 |
| 25 | *-* | 4930 | 1339 | 0932 |
| 26 | *-* | 4925 | 1340 | 0933 |
| 27 | *-* | 4920 | 1341 | 0934 |
| 28 | *-* | 4915 | 1342/1343 | 0934 |
| 29 | *virC1* | 4910 | - | 0935 |
| 30 | *-* | 4905 | - | - |
| 31 | *-* | 4900 | - | - |
| 32 | *-* | 4495 | - | - |
| 33 | *-* | 4890 | - | 0936 |
| 34 | *virB6* | 4885 | - | 0937 |
| 35 | *-* | 4880 | 1344 | 0938 |
| 36 | *-* | 4875 | 1345/1346 | 0939 |
|  | *-* | - | - | 0940 |
| 37 | *-* | 4870 | 1347/1348 | - |
| 38 | *-* | 4865 | 1349 | - |
| 39 | *-* | 4860 | 1350 | - |
| 40 | (*xer*) | 4855 | 1351 | 0941 |
| 41 | *virD2* | 4840 | 1353 | 0942 |
